# Periodic Fever, Aphthous Stomatitis, Pharyngitis and Cervical Adenitis (PFAPA) Syndrome in Iranian Children First Report of Iranian Periodic Fever and Autoinflammatory Registry (IPFAIR)

**Published:** 2014-10-24

**Authors:** Fatemeh Fereshteh Mehregan, Vahid Ziaee, Zahra Ahmadinejad, Fatemeh Tahghighi, Farah Sabouni, Mohamad-Hassan Moradinejad

**Affiliations:** 1 Children’s Medical Center, Pediatrics Center of Excellence; 2 Shahid Beheshti University of Medical Sciences; 3 Pediatric Rheumatology Research Group, Rheumatology Research Center; 4 Department of Pediatrics; 5 Department of Infectious Diseases, Tehran University of Medical Sciences, Tehran, Iran

**Keywords:** PFAPA syndrome, Periodic fever, Recurrent fever, Children, Autoinflammatory Disorders

## Abstract

***Objective:*** The periodic fever, aphthous stomatitis, pharyngitis and cervical adenitis (PFAPA) syndrome is a nonhereditary idiopathic febrile syndrome belonging to the group of autoinflammatory diseases. No longtime sequel was reported in this disease. Early diagnosis can lead physicians to treatment of this disorder with a short course steroid application and provide satisfaction of the patient’s family.

***Methods:*** This study is a prospective review of patients diagnosed with PFAPA syndrome who were registered in Iranian Periodic Fever and Autoinflammatory Registry (IPFAIR) through periodic fever clinic in the Children's Medical Center, Pediatric Center of Excellence in Tehran, Iran from January 2013 to March 2014.

***Findings:*** One hundred thirty patients were registered in our databases. Twenty-one (16.1%) patients including 15 males and 6 females had PFAPA. Normal growth was seen in all patients. The median age at onset was 18 months. The mean duration of fever was 4 days and the mean duration of intervals between fever episodes 21 days. Along with fever, all patients had characteristic symptoms. All patients were asymptomatic between fever episodes. Steroid was used in all patients and causing immediate reduction by 84.61%. Two patients received both steroid and colchicine because of their clinical feature and positive laboratory tests for PFAPA and familial Mediterranean fever. No patient received biological therapy or a tonsillectomy.

***Conclusion:*** The long diagnostic delay of PFAPA gives cause to concern indicating a need for greater awareness of the disease so that the diagnosis may be made timely.

## Introduction

Fever is not always a sign of infection. Autoinflammatory disorders (AIDs) are also characterized by relapsing fever episodes. One of the AIDs is periodic fever, aphthous stomatitis, pharyngitis and cervical adenitis (PFAPA) syndrome^[^^1^^]^. This syndrome was first described in 1987 as a sovereign clinical diagnosis^[^^2^^]^. PFAPA **b**elongs to a group of AIDs that is characterized by the fever episodes that seem to be unprovoked inflammations. Specific antibodies and antigens related to autoreactive T cells is not seen in AIDs unlike autoimmune diseases^[^^3^^]^**. **PFAPA syndrome is a sporadic idiopathic febrile syndrome. The onset of the disease is usually before the age of 5 years and associated with one or more symptoms such as aphthous stomatitis, pharyngitis and cervical adenitis. The disease is caused by the absence of respiratory infection and cyclic neutropenia^[^^2^^,^^4^^]^. Its etiology is unknown; no genetic mutation was detected. Recent studies indicate that T cell-regulated complement activation and production of IL-1 in PFAPA syndrome has changed and confirms the hypothesis that PFAPA syndrome is an autoinflammatory syndrome^[^^1^^]^. Currently, there are non-specific biological markers that may help with the diagnosis^[^^5^^]^. To diagnose this disease, it is necessary that be ruled out the other periodic fever that may overlap clinically with PFAPA syndrome^[^^6^^,^^7^^]^. Although no long-term complications of this syndrome^[^^8^^,^^9^^] ^has been reported, it has a major impact on patients and is also a cause of concern for families. Early diagnosis of the disease provides the ability to control disease symptoms and family comfort, and prevents frequent hospitalization as well as use of unnecessary antibiotics^[^^10^^]^. 

 The aim of this study was to describe the clinical manifestations PFAPA syndrome from patients who were registered in Iranian Periodic Fever and Autoinflammatory Registry (IPFAIR) at Children's Medical Center, which is established for this purpose since June 2013.

## Subjects and Methods

This investigation is a prospective cross sectional descriptive study of patients diagnosed with PFAPA syndrome registered in the autoinflamatory disorders system registry by Periodic Fever Clinic at Children's Medical Center, Pediatrics Center of Excellence in Tehran, Iran from June 2013 to March 2014. 

 A total of 130 patients were recorded as periodic fever syndrome in the registration database. All patients have been enrolled in the Iranian Periodic Fever and Autoinflammatory Registry (IPFAIR) based on algorithmic approach that we published before ^[^^11^^,^^12^^]^. All patients fulfilled the PFAPA syndrome criteria according to Eurofever classification^[^^13^^]^ were enrolled in this study. These criteria include: 

1) Fever for a period of at least 6 months, the daily minimum temperature 38.5°C for 2-7 days 

2) Pharyngitis, cervical adenitis, and/or canker sores 

3) Exclusion of other causes of periodic fever (by family history and the absence of typical clinical features and laboratory markers) 

4) Ruling out infections, immunodeficiency and cyclic neutropenia 

5) Completely asymptomatic interval periods and normal linear growth.

 In all patients, monogenic hereditary periodic fever syndromes were ruled out. Study of gene mutations was performed in cases in which it was necessary based on positive family history or clinical suspicious to familial Mediterranean fever (FMF). 

 Infection was ruled out by throat culture, blood and urine culture. Immunodeficiency disorders was ruled out by measuring immunoglobulin levels, leukocyte count and further immunological investigation was carried out if necessary.

 Cyclic neutropenia was ruled out by measuring levels of neutrophils during episodes of fever and in the intervals between episodes. Any patient, whose neutrophil count was less than 1.5˟10^⁹^ each time, was excluded from the study.

 In addition to demographic and anthropometric characteristics, the following data were recorded for each patient: duration of fever episodes, the interval between episodes of fever, clinical symptoms including fever, pharyngitis (exudative, erythematous), cervical adenitis, aphthous stomatitis and other symptoms and response to treatment. Laboratory findings for leukocytosis, C-reactive protein (CRP), and erythrocyte sedimentation rate (ESR) were recorded during episodes of fever and afebrile periods.

 Data analysis was performed using two levels of descriptive and analytical statistics. In descriptive statistics, statistical characteristics of frequency deviation, minimum and maximum average data were analyzed. For analytical data chi-square test and t-test was used. The data collected were analyzed using the SPSS program. *P* values less than 0.05 were considered statistically significant. 

**Table1:** Demographic and clinical characteristic of attacks patients with PFAPA syndrome

**Characteristic**	**median**	**Range**	**mean**	**Confidence Interval**
**Age at onset (mo)**	18	6-112	32.47	17.05-47.86
**Age at diagnosis (mo)**	55.5	17-163	66.10	46.70-85.49
**Duration between onset disease to diagnose (mo)**	18	2-130	33.68	17.51-49.86
**Attack duration (days)**	4.45	2-8		
**Interval between attacks (days)**	21	14-60		

PFAPA: Periodic fever, aphthous stomatitis, pharyngitis and cervical adenitis

## Findings

From 130 patients with periodic fever, a total of 28 (21.5%) patients, 7 patients (6 patients because of incomplete period of the disease and 1 patient being older than 18 years) were excluded. Finally 21 (16.1%) patients were studied of which 15 were males and 6 females. All (100%) patients had normal growth. The age at onset was less than 5 years (78.9%). Median age at onset of symptoms was 18 months and median age at diagnosis was 55 months.

 Mean length of duration of fever episodes was 4.5 days and the mean time between attacks of fever episodes 24.74 days (95% CI=21.20-28.28), respectively. The mean interval between onset of symptoms to diagnosis was 33.68 months (95% CI=17.51-49.86) (Table 1).

 Our patients had characteristic symptoms of pharyngitis (100%), lymphadenopathy, cervical adenitis (61.90%) or aphthous stomatitis (42.85%) with fever at the same time. The patients had other symptoms such as vomiting, abdominal pain, headache, weakness, fatigue, myalgia that are shown in Table 2. Only 23.80% of patients had all the 3 characteristic symptoms of PFAPA syndrome. 

 We found leukocytosis (71.42%), polynucleosis (90%), elevated ESR (84.61%), elevated CRP (92.30%) during the early episode of fever in patients who were tested. 6 patients with positive genetic test of familial Mediterranean fever (FMF) had clinical symptoms of PFAPA syndrome, and responded to treatment with steroid; 2 patients had clinical symptoms of both syndromes and were treated with colchicine, They were showing signs of PFAPA syndrome (high fever, sore throat, lymphadenopathy) from the age of 4 to 6 years who responded to steroids. Their symptoms included fever, vomiting, and abdominal pain which recurred when colchicine was discontinued so that colchicine had to be reapplied. Serum immunoglobulin levels were normal in all patients.

 Some (66.8%) of the patients had a history of frequent hospitalizations because of recurrent fever, leukositosis, pharyngitis or oral ulceration (1 to 10 admissions). Approximately 81.25% of patients had received penicillin or other antibiotics and 37.5 % had received steroids before admission.

**Table2:** Distribution of sign and symptom associated with the fever episode in PFAPA patients

**Associated Features ** **with Fever symptom**	**Number (%)**	**Associated Features with ** **Fever sign**	**Number (%)**
**Fatigue**	8/21 (38.1)	**Rash**	1/21 (4.8)
**Abdominal pain**	9/21 (42.8)	**Pharyngitis**	21/21 (100)
**Nausea and vomiting**	10/21 (47.6)	**Aphthous stomatitis**	9/21 (42.8)
**Headache**	9/21 (42.8)	**Genitalia aphthomatous**	0/24 (0)
**Oral aphthous**	9/21 (42.8)	**Lymphadenopathy**	13/21 (61.9)
**Sore throat**	21/21 (100)	**Abdominal tenderness**	0/2 (0)
**Paleness**	2/21 (9.5)	**Arthritis**	1/2 (4.8)
**Chest pain**	1/21 (4.8)		
**Joint pain**	5/21 (23.8)		
**Myalgia**	5/21 (23.8)		

PFAPA: Periodic fever, aphthous stomatitis, pharyngitis and cervical adenitis

 Steroids (prednisolon or dexamethasone) were administered in 21 patients after diagnosis, leading to reducion of fever in 84.61% of the patients. Moreover 2 patients had received cimetidine and 2 other colchicine in addition to steroids. None of the patients had undergone a tonsillectomy and we have not used biological agents in our patients up to now. Six patients had a positive genetic test for FMF and clinical symptoms of PFAPA syndrome.

## Discussion

This prospective study was done on patients with PFAPA syndrome, the clinical manifestations of which were recorded in the autoinflammatory database registration. Our results indicating median age at onset of PFAPA was 18 (6-112) months, this is similar to results found in other studies, namely 18 (1-60) months, 12 (2-60) months, 22 (1-120) months^ [^^6^^,^^14^^,^^15^^]^, and it is different from the results of the studies by Thomas et al (2.8 years)^[^^9^^]^ and Feder and Salazar (39.6 months)^[^^14^^]^ who found a much higher median age at onset. The mean duration of febrile episodes of 4 days and the mean duration of intervals between febrile episodes of 21 days was similar to other studies^[^^2^^,^^9^^,^^10^^,^^15^^,^^16^^]^, in which mean duration of febrile episodes was 4-5 days and the mean duration of interval between febrile episodes 30 days.

 The patients had fever plus at least one of the following characteristic clinical features: pharyngitis, aphthous stomatitis or cervical adenitis. Clinical manifestations are different in various studies, so all symptoms are not necessary for the diagnosis of this syndrome ^[^^10^^]^.

 In our study, pharyngitis (100%), cervical adenitis (61.9%) and aphthous stomatitis (42.8%) were found as the most common clinical manifestation. Pharyngitis in other studies (65-96%), aphthous stomatitis (36-67%), cervical adenitis (61-77%) have been reported^[^^6^^,^^9^^,^^15^^,^^16^^]^. Kyvsgarad et al reported cervical adenitis in 96.8%^[^^10^^]^. 

 The prevalence of other symptoms in the study of Kyvsgarad et al^[^^10^^]^ was much higher than in other studies(96.8%). Fever with abdominal pain and headache in 18-65%^[^^6^^,^^9^^,^^15^^-^^17^^]^, vomiting in 27-30%^[^^7^^,^^16^^]^, and diarrhea in 29-30%^[^^6^^,^^9^^]^ of patients were reported. In our study, the most common symptoms associated with fever were vomiting (56.25%), abdominal pain (43.75%), headache and fatigue (37.5%), myalgia (18.75%), arthralgia and pallor (12.5%) and chest pain (6.25%).

 We have used steroids (dexamethasone or prednisone) to treat patients. After the use of steroids (prednisone or dexamethasone) fever was reduced by 84.61% (19/21) of patients. In a study of Kyvsgarad et al^[^^10^^]^ also reducing of fever with steroids therapy was reported in 87.5%. In another study of 72 patients that were treated with prednisone reducing fever was reported in 97%^[^^16^^]^.

 Oral prednisone alone effects on symptoms and improves them; however, it causes shortening of the intervals between attacks and also does not forestall the onset of fever. Nevertheless, it is very important to control fever in certain circumstances^[^^10^^]^. 

 We accept some limitation, because of the short duration of the data recording system startups. Some patients who did not fill 6 months of the disease, (which is necessary for the confirmation of the syndrome) were not enrolled in this study. However, despite the low number of patients, the results of our study were similar to those of many other studies.

## Conclusion

Although PFAPA syndrome was first described 20 years ago and the diagnostic process has been reduced over time, the delay in diagnosis leads to frequent hospitalizations and use of antibiotics and other useless medicines, that is a cause for family concern and points to a need for greater awareness of the disease. Living with PFAPA syndrome is really annoying. Early diagnosis and early appropriate intervention is the key to getting rid of this condition.
